# Regulation of Flower Bud Differentiation Hormones and Identification of Related Key Genes in *Dendrobium officinale* Based on Multi-omics Analysis

**DOI:** 10.3390/plants14172668

**Published:** 2025-08-27

**Authors:** Zhihao Yin, Daoliang Yan, Jianke Du, Chongbo Sun

**Affiliations:** 1 Innovation Center of Chinese Medicinal Crops, Horticulture Institute, Zhejiang Academy of Agricultural Sciences, Hangzhou 310021, China; 2 State Key Laboratory of Subtropical Silviculture, Zhejiang A & F University, Hangzhou 311300, China; liangsie@zafu.edu.cn; 3 Jiangsu Key Laboratory for Conservation and Utilization of Plant Resources, Institute of Botany, Jiangsu Province and Chinese Academy of Sciences (Nanjing Botanical Garden, Men. Sun Yat-sen), Nanjing 210014, China

**Keywords:** *Dendrobium officinale*, flower bud differentiation, 6-BA, GA_3_, transcriptome, metabolome

## Abstract

*Dendrobium officinale,* an orchid of significant medicinal and ornamental value, exhibits poorly characterized hormonal regulation of flower bud differentiation. To address this knowledge gap, we employed an integrated multi-omics approach combining physiological, transcriptomic, metabolomic, and network analyses to elucidate the molecular mechanisms underlying the coordinated action of 6-Benzylaminopurine (6-BA) and Gibberellin A3 (GA_3_) in this critical developmental process. Our key findings reveal that combined 6-BA and GA_3_ treatment significantly enhances flower bud differentiation and induces stage-specific fluctuations in soluble sugar, protein, and starch levels. Transcriptomic profiling identified 11,994 differentially expressed genes (DEGs), with DEGs specific to the hormone-treated stage showing pronounced enrichment in plant hormone signal transduction and plant–pathogen interaction pathways. Metabolomic analysis uncovered 18 stage-specific differential metabolites (DAMs) during hormone treatment, including GA_3_, 6-BA, and OPDA, whose accumulation dynamics were strongly correlated with the progression of differentiation. Weighted gene co-expression network analysis (WGCNA) pinpointed key hub genes within the yellow module, notably transcription factors from the *C2H2*, *bZIP*, and *NAC* families. Their interaction network demonstrated significant correlation with the transcriptional regulation of hormone-responsive genes. Significantly, this study establishes the first molecular framework for 6-BA and GA_3_ regulation of flower bud differentiation in *D. officinale*. We demonstrate a metabolomic–transcriptomic coordination network driven by these hormones, where key hub genes form regulatory modules with transcription factors. Dynamic shifts in endogenous hormones reinforce the flowering signal. These findings provide crucial molecular targets for precision flowering control and molecular breeding strategies in orchids.

## 1. Introduction

Orchids (family Orchidaceae) comprise approximately 700 genera and 28,000 species globally, distributed across terrestrial ecosystems worldwide except polar regions and hyper-arid deserts, with peak diversity in tropical zones [1,2,3,4]. Commercial orchid production constitutes a significant economic sector in horticulture. Chinese breeders have developed world-class cultivars from wild orchids, including *Paphiopedilum*, *Cymbidium*, and *Dendrobium*. *D. officinale*, prevalent among these, primarily inhabits southwestern China and Jiangnan regions, containing medicinally valuable compounds including polysaccharides, flavonoids, phenolics, and amino acids [5]. Its flowers and stems serve as primary nutrient reservoirs and key harvest organs. However, natural cultivation yields low flower bud differentiation rates, and the mechanisms regulating flowering remain undefined, which constrains scalable production.

Flower bud differentiation marks the vegetative-to-reproductive transition and represents the pivotal developmental phase in flowering plants. This process integrates exogenous and endogenous cues. In *Arabidopsis thaliana*, floral development engages six pathways: photoperiod, vernalization, ambient temperature, gibberellin, autonomous, and age pathways [6]. These pathways converge through integrator genes, including *FLOWERING LOCUS T* (*FT*), *LEAFY* (*LFY*), and *SUPPRESSOR OF OVEREXPRESSION OF CONSTANS 1* (*SOC1*), to regulate flowering [7]. Orchid floral development genes predominantly regulate flower organogenesis and flowering time. In Cymbidium goeringii, the SOC1 homolog *CgSOC1* (MADS-box) regulates floral development and organ formation [8]. *Phalaenopsis SEPALLATA-like* (*SPL*) genes critically regulate floral organogenesis [9]. Meanwhile, the *MADS6* gene was isolated and identified in Phalaenopsis. It is a *GLOBOSA/pistillata-like* gene involved in the formation of floral-like structures [10]. Heterologous expression of the *FT* homolog *DoFT1* from *Dendrobium ochreatum* accelerates flowering in *tobacco* [11]. In *Arabidopsis* thaliana, overexpression of the homologous gene *DnAGL19*, which is similar to *SOC1/TM3-like*, can regulate the expression of *HOS1-FT* in *Arabidopsis*, thereby promoting or inhibiting the transition to flowering [12].

Plant growth regulators, notably cytokinin (e.g., 6-BA) and gibberellin (e.g., GA_3_), are widely used in horticulture to modulate flowering [13,14]. Crucially, combined applications of 6-BA and GA_3_ often enhance flower bud differentiation more effectively than single-hormone treatments across diverse plant species (Table 1). 

Despite this empirical evidence and the economic importance of *D. officinale*, current research predominantly describes physiological responses to these hormones, leaving a significant gap in understanding their coordinated molecular mechanisms.

Based on the superior efficacy of combined hormone treatments observed in other systems, we hypothesized that 6-BA and GA_3_ act synergistically to promote flower bud differentiation in *D. officinale* through a coordinated molecular network involving key genes and metabolites. To test this hypothesis and address the knowledge gap, we systematically investigated the molecular mechanisms underlying 6-BA and GA_3_ coordination using integrated physiological, transcriptomic, metabolomic, and weighted gene co-expression network analyses (WGCNA). This study aims to elucidate the molecular framework of hormone-regulated flower bud differentiation in *D. officinale*, providing critical targets for precision flowering control and molecular breeding.

## 2. Results

### 2.1. Morphological and Physiological Changes in D. officinale Flower Buds Under Different Hormone Treatments

**Figure 1:**
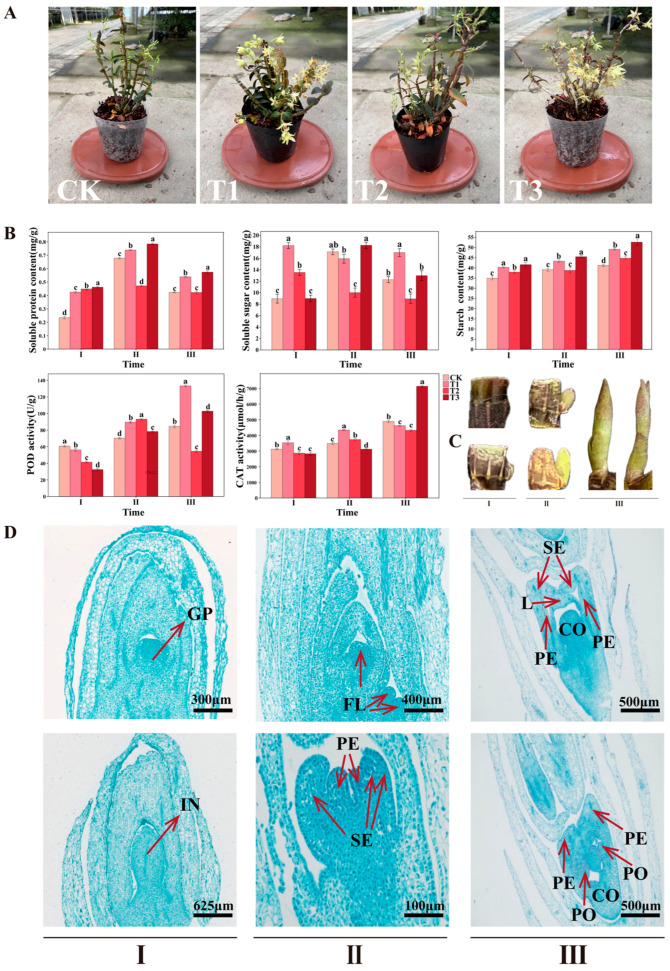
Phenotypic and physiological changes under different treatments. (**A**) Phenotypes of *D. officinale* under treatments. (**B**) Measurements of physiological parameters. (**C**) Morphology of *D. officinale* flower buds at different stages. (**D**) Cellular structures observed under optical microscopy. Note: (**A**) Different lowercase letters indicate significant differences (*p* < 0.05) between different treatment groups, and the error bars represent the standard deviation. (**D**) GP—growth cone; IN—inflorescence primordium; FL—flower bud primordium; SE—sepal primordium; PE—petal primordium; CO—column primordium; L—Lip; and PO—pollinium.

We performed phenotypic analysis of *D. officinale* under hormone treatments before conducting transcriptome studies (Figure 1A). All treatments were evaluated at stage III (90 days post-initial treatment). Compared with CK (water treatment), the three treatments T1 (6-BA 200 ppm), T2 (GA_3_ 50 ppm), and T3 (6-BA 200 ppm + GA_3_ 50 ppm) all had the effect of advancing the flowering period. In terms of the number of flowers, T3 > T1 > T2 > CK. In the T2 treatment, leaves gradually turned yellow and fell off during the treatment, but the plants still flowered normally and earlier than the control. We quantified dynamic physiological changes during early bud differentiation by measuring soluble protein, soluble sugar, starch, peroxidase (POD), and catalase (CAT) activities in leaves across three developmental stages. All physiological parameters exhibited stage-dependent variation during bud differentiation (Figure 1B). In treatment T3, soluble protein and sugar levels were highest at stage II and decreased by stage III. Notably, T3 maintained higher levels of soluble protein and starch compared with the other treatments. Conversely, T3’s POD activity was consistently lower than in T1, but it increased over time, peaking at stage III. All treatments showed consistent starch accumulation over time. For CAT activity, T3 values were lower than the control in stages I and II but peaked in stage III. At that peak, CAT activity in T3 was 39.8% and 36.4% higher than the control in stages II and III, respectively. Collectively, the 6-BA 200 ppm + GA_3_ 50 ppm combination represents the most effective regime among tested treatments for promoting *D. officinale* bud differentiation.

### 2.2. Transcriptome Quality Control and Differential Gene Clustering Analysis

**Figure 2:**
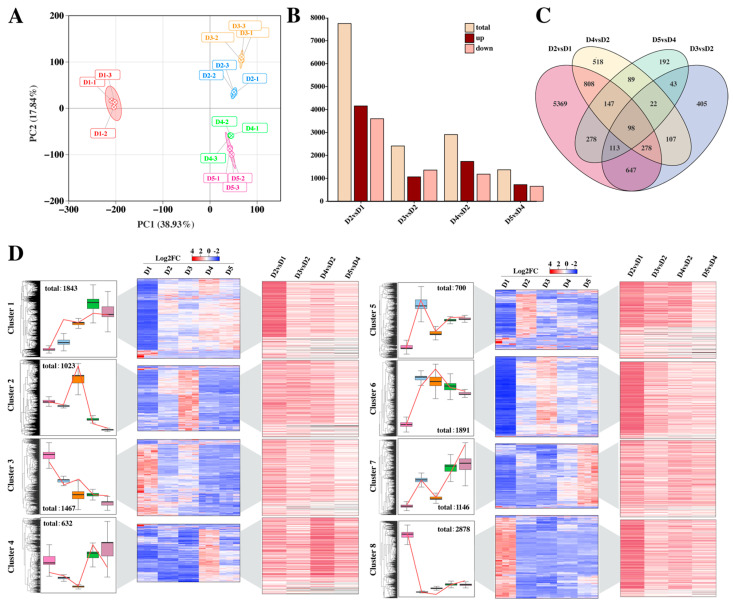
(**A**) Principal component analysis (PCA). (**B**) The number of upregulation and downregulation of differential genes. (**C**) Venn graph under hormone treatment. (**D**) Differential gene clustering analysis. Note: (**D**) In the box plot, pink, blue, orange, green and purple respectively represent Group D1, D2, D3, D4 and D5.

In this study, we constructed transcriptome libraries across five developmental stages (treatment and control groups) and generated 119.94 Gb of high-quality clean reads. All samples yielded >7 Gb clean reads with Q30 > 93% (proportion of bases with Phred score ≥ 30), confirming data reliability (Table S1). Alignment to the *D. officinale* reference genome achieved >90% efficiency per sample, validating data integrity. PCA revealed clear separation among developmental stages in principal component space, with PC1 and PC2 explaining 38.93% and 17.84% of variance, respectively (Figure 2A). This demonstrates significant heterogeneity in stage-specific expression profiles, enabling the robust identification of differentially expressed genes. We used DESeq2 to identify DEGs between developmental stages, applying a threshold of |log2(fold change)| ≥ 1 and adjusted *p*-value < 0.05. The results showed that the expression trends of DEGs varied in different stages. In the comparisons of D2 vs. D1, D3 vs. D2, D4 vs. D2, and D5 vs. D4, 7738, 1713, 2067, and 982 DEGs were identified, respectively. Among them, 4147, 750, 1233, and 518 DEGs showed increased expression, while 3591, 963, 834, and 982 DEGs showed decreased expression (Figure 2B). Venn analysis identified 147 co-expressed DEGs in the D2 vs. D1, D4 vs. D2, and D5 vs. D4 comparisons (Figure 2C). Specifically, D3 vs. D2 contained 405 uniquely expressed genes (Figure 2C). These genes are likely closely involved in the hormone regulation of flower bud differentiation. Using the k-means clustering method, the DEGs from the five stages (D1–D5) were grouped into eight clusters (Figure 2D). Among them, the DEGs in Cluster 5 were significantly activated in the D2 stage, which may be related to the early initiation of flower bud differentiation; the DEGs in Cluster 2 and Cluster 6 were specifically upregulated in the D3 stage, and these genes may be the key genes activated under hormone stimulation; the DEGs highly expressed in the D4 and D5 stages were clustered in Cluster 4 and Cluster 7. This clustering analysis not only revealed the temporal characteristics of gene expression but also provided clues for the exploration of key regulatory nodes.

### 2.3. Annotation of DEGs

**Figure 3:**
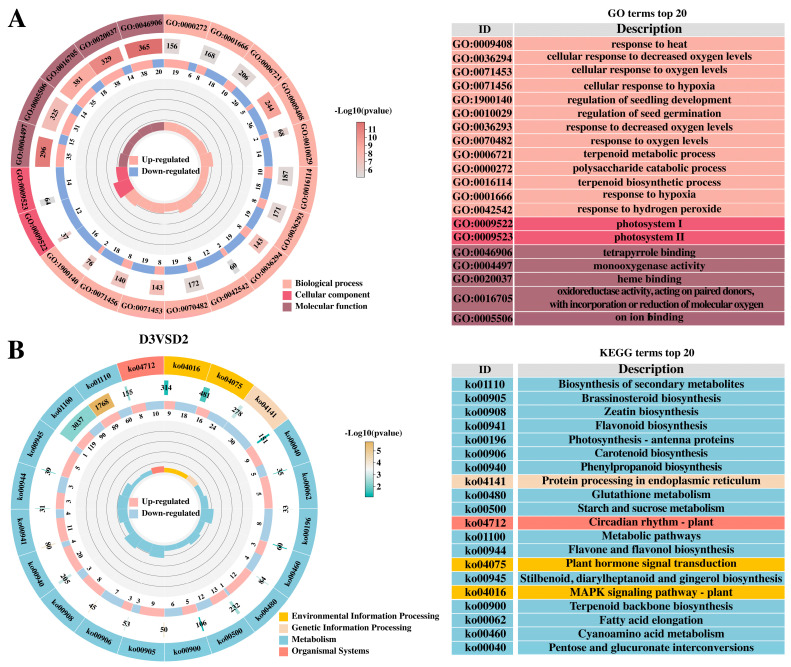
(**A**) GO analysis of DEGs between D3 and D2. (**B**) KEGG analysis of DEGs between D3 and D2.

We performed GO and KEGG enrichment analyses for DEGs from D3 vs. D2, D2 vs. D1, D4 vs. D2, and D5 vs. D4 comparisons. The gene ontology (GO) framework comprises three domains: biological process (BP), cellular component (CC), and molecular function (MF). The GO enrichment results showed that the 935, 4514, 1245, and 525 genes were annotated as BP, CC, and MF, respectively. For all the upregulated and downregulated genes in the comparisons of D2 with D1, D4 with D2, and D5 with D4, the top ten annotated GO terms were all cellular anatomical entity (GO: 0110165), cellular process (GO: 0009987), binding (GO: 0005488), metabolic process (GO: 008152), catalytic activity (GO: 0003824), response to stimulus (GO: 0050896), biological regulation (GO: 0065007), regulation of biological process (GO: 0050789), developmental process (GO: 0032502), and multicellular organismal process (GO: 0032501). In the top ten GO terms of D2 vs. D1, D4 vs. D2, and D5 vs. D4, 3679, 2700, 2551, 2349, 2069, 1374, 1235, 1137, 841, and 694 were annotated as upregulated genes, and 2907, 2175, 2175, 1891, 1654, 1288, 1024, 947, 580, and 523 were annotated as downregulated genes (Figure S1). The GO enrichment analysis of the D3 vs. D2 comparison revealed that the DEGs identified after hormone treatment were mainly enriched in the following GO terms: in the biological process category—response to heat, cellular response to decreased oxygen levels, cellular response to oxygen levels, cellular response to hypoxia, regulation of seedling development, regulation of seed germination, etc.; in the cell composition category—photosystem I and photosystem II; in the cell organization category—tetrapyrrole binding, monooxygenase activity, heme binding, oxidoreductase activity, acting on paired donors, with incorporation or reduction of molecular oxygen, and iron ion binding (Figure 3A).

The DEGs were further classified by the Kyoto Encyclopedia of Genes and Genomes (KEGG) database in terms of metabolism. The advantage of this approach is that the pathway significance enrichment analysis is conducted based on the pathways in the KEGG database, using the hypergeometric test to identify the pathways that are significantly enriched in the DEGs compared to the entire genomic background. In the D3 vs. D2 comparison, a total of 441 DEGs were assigned to 115 KEGG pathways. We listed the top 20 pathways based on the *p*-value (*p* < 0.05), among which most of the DEGs were assigned to the “Metabolic Pathways” and “Secondary Metabolite Biosynthesis Pathways”, respectively, enriching 149 and 209 DEGs. The plant–pathogen interaction pathway and the plant hormone signal transduction pathway were also ranked second. The plant hormone signal transduction pathway included tryptophan metabolism, zeatin biosynthesis, diterpenoid biosynthesis, and carotenoid biosynthesis (Figure 3B). Interestingly, in the comparisons of D2 vs. D1, D4 vs. D2, and D5 vs. D4, a large number of DEGs were also enriched in the plant hormone signal transduction pathway, with 213, 61, and 32 DEGs, respectively (Figure S2). This indicates that the plant hormone signal transduction pathway plays an important regulatory role in flower bud differentiation, coordinating the dynamic balance of endogenous hormones with external environmental signals to drive the transition of plants from vegetative growth to reproductive growth. Additionally, in the comparisons of D2 vs. D1, D4 vs. D2, and D5 vs. D4, most of the DEGs were also enriched in the MAPK signaling pathway—plant and phenylpropanoid biosynthesis, and a few DEGs were enriched in the phenylpropanoid biosynthesis and plant circadian rhythm pathways. These results indicate that flower bud differentiation is a complex process involving multiple biological pathways.

### 2.4. Transcription Factor Analysis

**Figure 4:**
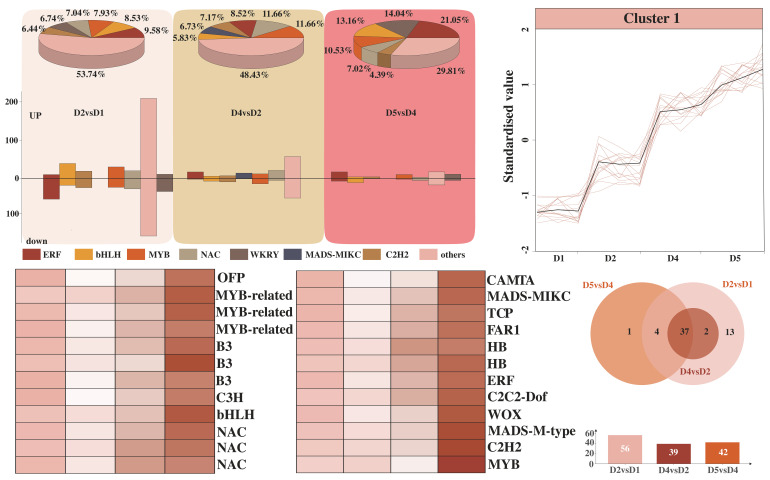
Proportion and number of transcription factors in D2 vs. D1, D4 vs. D2, and D5 vs. D4 (**A**). Transcription factors with continuously increasing expression levels (**B**) and expression analysis (**C**). Venn diagram of transcription factors (**D**).

A large number of studies have confirmed that transcription factors (TFs) are involved in and significantly influence the molecular regulatory network of flower bud differentiation. In this study, we detected 668, 223, and 114 differentially expressed TFs in the comparisons of D2 vs. D1, D4 vs. D2, and D5 vs. D4, respectively, including gene families such as *ERF*, *bHLH*, *MYB*, *NAC*, *WKRY*, and *C2H2* (Figure 4A). Among them, *ERF* had the largest proportion, accounting for 9.58%, 8.52%, and 21.05%, respectively. In the comparisons of D2 vs. D1, D4 vs. D2, and D5 vs. D4, we identified 338 upregulated and 330 downregulated, 134 upregulated and 89 downregulated, and 66 upregulated and 48 downregulated TFs, respectively. Subsequently, we screened out 24 TFs with continuously increasing expression levels from these differentially expressed TFs (Figure 4B). Among them, *MYB-related*, *NAC*, and *B3* were the most numerous, each with three, and the rest of the TFs were one to two in number. It is speculated that these TFs may be closely related to flower bud differentiation (Figure 4C). In addition, we identified 56, 39, and 42 gene families in the comparisons of D2 vs. D1, D4 vs. D2, and D5 vs. D4, respectively (Figure 4D). Among them, 41 gene families were shared by all three groups; 1 was unique to D5 vs. D4, which was *C2C2-Dof*; 4 gene families were shared by D5 vs. D4 and D2 vs. D1, namely *B3-ARF*, *GARP-ARR-B*, *HB-KNOX*, and *TUB*; and 2 gene families were shared by D2 vs. D1 and D4 vs. D2, namely *EIL* and *LLFY*.

### 2.5. WGCNA Screening of Transcription Factors Regulating D. officinale Flower Bud Differentiation by Hormones

**Figure 5:**
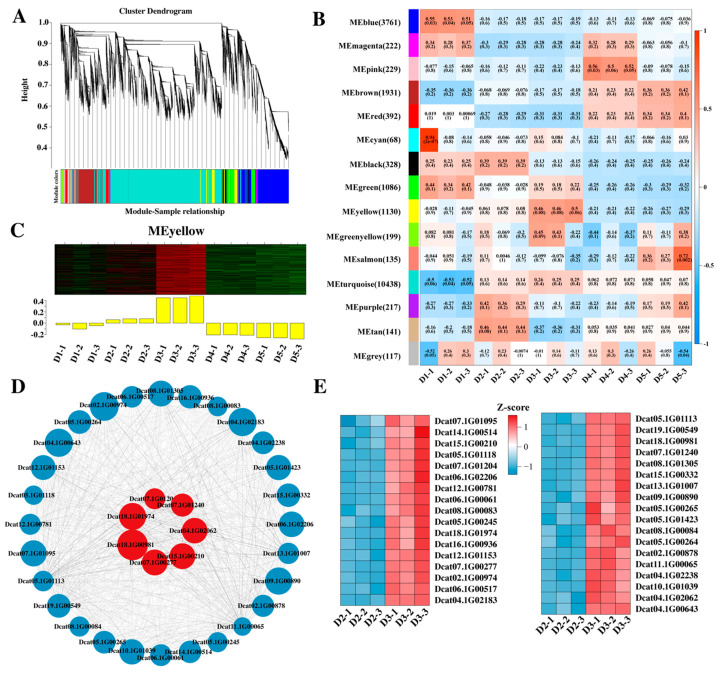
WGCNA analysis of genes related to flower bud differentiation in *D. officinale* (**A**–**C**). The yellow module shows the gene-related network connected to the C2H2 (Dcat18.1G00981) gene with an edge weight of >0.40 (**D**), and the expression analysis of these genes (**E**). Note: (**A**) Each color in the figure represents that all the genes corresponding to each color on the clustering tree belong to the same module. (**B**) Each number in the grid represents the correlation between the module and the sample. The closer the value is to 1, the stronger the positive correlation between the module and the sample. The number in parentheses represents the significance *p*-value. The smaller the value, the stronger the significance. (**D**) The larger the edge weight, the darker the color of the line connecting the two points. The higher the connectivity, the larger the node. Red represents transcription factors.

This study employed hormone treatment and control groups of 15 samples from five stages of flower bud differentiation in *D. officinale* to conduct WGCNA. We evaluated correlations between gene expression changes across stages and sample traits to identify transcription factors that might regulate flower bud differentiation in *D. officinale.* Firstly, WGCNA was used to analyze the expression patterns of 20,394 DEGs, which were classified into 15 coexpression modules based on their expression patterns (Figure 5A,B). To explore transcription factors related to hormone regulation, the correlation between the expression patterns of each module and the D3 stage was further analyzed. The results showed that the “yellow” module was highly correlated with the D3 stage after hormone treatment (Figure 5C). A total of 1130 genes were identified in this module, and the top 50 genes with the highest connection strength were considered hub genes. Among these 50 hub genes, 10 transcription factors were found, including *bZIP* (Dcat11.1G00701), *C2H2* (Dcat18.1G00246, Dcat18.1G00825, Dcat18.1G00981, Dcat18.1G01974), *GRAS* (Dcat10.1G00415), *NAC* (Dcat10.1G00058), *NF-YC* (Dcat11.1G00886), *TCP* (Dcat16.1G00059), and *TUB* (Dcat18.1G00184). As hub genes, these transcription factors may play important roles in the hormone-regulated process of flower bud differentiation.

To further explore other potential transcription factors involved in hormone regulation, genes related to the hub gene *C2H2* (Dcat18.1G00981) with edge weights > 0.40 in the “yellow” module were selected for analysis. The results indicated that 35 genes were regulated by *C2H2* (Dcat18.1G00981), including the *AP2/ERF-ERF* family (Dcat04.1G02062), *B3-ARF* family (Dcat07.1G00277), *C2C2-Dof* family (Dcat15.1G00210), *C2H2* family (Dcat18.1G01974), *GARP-ARR-B* family (Dcat07.1G01204, Dcat07.1G01240), and other non-hub transcription (Dcat18.1G00981) factor genes related to *C2H2* (Figure 5D). All were significantly upregulated after combined 6-BA and GA_3_ treatment, indicating a close role in hormone-induced flower bud differentiation (Figure 5E).

### 2.6. Metabolomics Analysis

**Figure 6:**
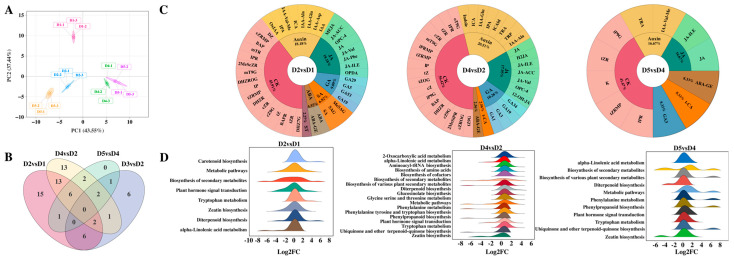
Metabolomics PCA (**A**). Venn diagram of different differential groups (**B**). Number of endogenous hormones in different differential groups (**C**). KEGG analysis of different differential groups (**D**). Note: the higher the mountain peak, the more differential metabolites are enriched in this pathway. The color has no special meaning.

We profiled hormone metabolomes across five bud differentiation stages (D1–D5) under natural and treated conditions. PCA revealed clear inter-stage separation, confirming data reliability (Figure 6A). Venn diagrams illustrate comparative relationships. We identified six shared metabolites in D2 vs. D1, D4 vs. D2, and D5 vs. D4, and six unique metabolites in D3 vs. D2. All of these were related to cytokinins (CK) and gibberellins (GA) (Figure 6B). Subsequently, we performed Krona analysis on the identified DAMs to illustrate the distribution of metabolite classes. The results showed that 44, 39, and 12 DAMs were identified in the comparisons of D2 vs. D1, D4 vs. D2, and D5 vs. D4, respectively. Among them, CK accounted for the largest proportion—45.91%, 56.42%, and 41.67%—followed by auxins (Auxin) and jasmonic acid (JA). Notably, no strigolactone (SL) was detected in the later stages of bud differentiation or flower development, suggesting that SL may act only in the early and middle stages (Figure 6C). KEGG enrichment analysis showed that the DAMs were significantly associated with hormone biosynthesis pathways (zeatin, carotenoid, α-linolenic acid, tryptophan, and diterpenoid biosynthesis), confirming tight hormonal regulation of differentiation (Figure 6D).

**Figure 7:**
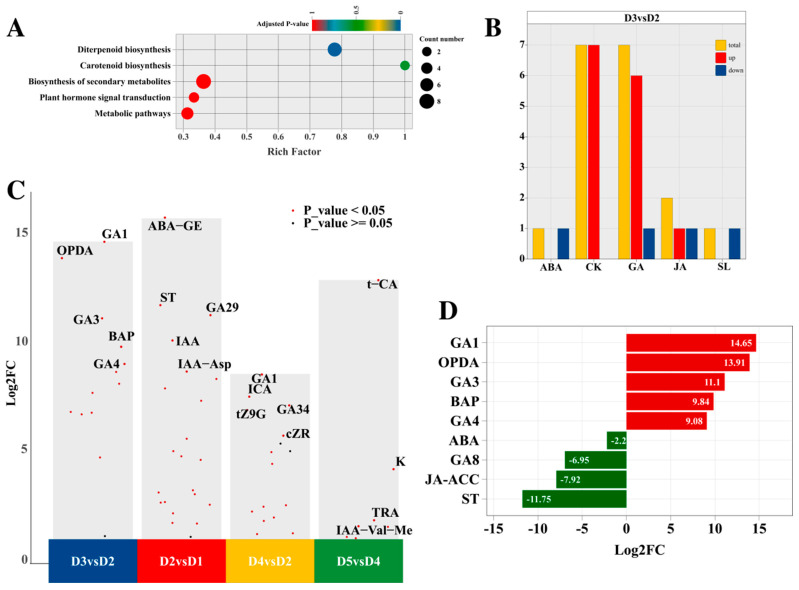
KEGG analysis (**A**), endogenous hormone quantity (**B**), differential results of the different groups (**C**), and the differential ratios of the DAMs (**D**) in the D3 versus D2 comparison group. Note: (**D**) Red color indicates that log_2_FC > 0, while green color represents that log_2_FC < 0.

Given that combined 6-BA and GA_3_ treatment enhanced bud differentiation, we analyzed the hormone-treated stage (D3) versus its control (D2) to elucidate the coordination mechanism. KEGG enrichment highlighted diterpenoid biosynthesis and carotenoid biosynthesis (Figure 7A), indicating hormone treatment activates these pathways. This forms a dual regulatory network integrating hormone coordination and metabolic homeostasis, promoting cell proliferation, differentiation, and environmental adaptation during mid-differentiation. Plant hormone signal transduction enriched ABA, GA_1_, and GA_4_, suggesting that exogenous hormones promote differentiation by coordinating ABA and active GAs. Eighteen DAMs were identified in D3 vs. D2, with CKs and GAs being most abundant (seven each) (Figure 7B). Hormone treatment significantly increased metabolites, including BAP, GA_1_, GA_3_, and GA_4_. Intriguingly, GA- and CK-related metabolites (GA_29_, GA_34_, tZ9G, and K) increased during mid- and late-stage differentiation and flower development, underscoring their importance (Figure 7C). Top upregulated DAMs (by fold-change) included the JA metabolite 12-Oxophytodienoic acid (OPDA), suggesting synergistic promotion with CKs and GAs. The top downregulated DAMs were ABA, GA_8_, Jasmonate-1-aminocyclopropane-1-carboxylic acid (JA-ACC), and Strigol (ST) (Figure 7D).

### 2.7. Differential Metabolite Clustering Analysis

**Figure 8:**
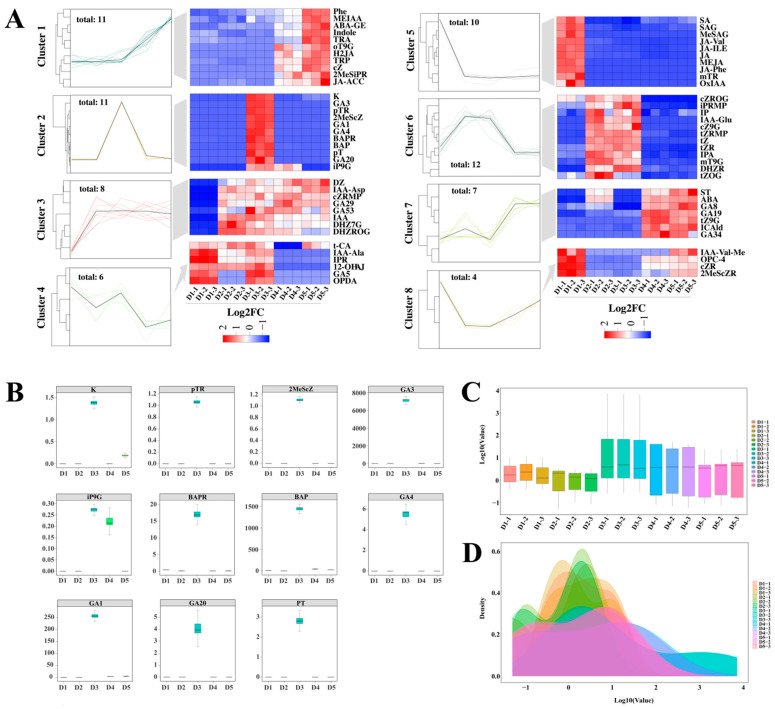
Clustering analysis of DAMs (**A**). Significance analysis of DAMs related to hormone treatment (**B**). Box plot of expression levels (**C**). Density distribution graph of expression levels (**D**). Note: (**A**) Different colors represent different categories.

Performing cluster analysis of the DAMs across the five stages using heat maps of expression patterns revealed eight distinct clusters (Figure 8A). Among them, the metabolites in Cluster 5 were specifically upregulated in stage D3, suggesting that these compounds play a key role in initiating flower bud differentiation. Metabolites in Cluster 6 increased significantly in D2, and their expression pattern closely resembled that of stage D3 after hormone treatment. This suggests that these compounds might not be directly involved in the hormone-mediated promotion of differentiation. Further analysis revealed that this cluster was mainly composed of cytokinins, with their expression peaking only in the mid-stage of differentiation. This indicates that CKs might specifically regulate this developmental stage. In contrast, the metabolites in Cluster 7 were consistently highly expressed from the late stage of bud differentiation through flower development, with gibberellins being the most abundant. This suggests that GA may dominate developmental regulation during this period. Interestingly, 11 DAMs were identified in Cluster 3 throughout the flower bud differentiation cycle, and these metabolites showed specific high expression patterns after hormone treatment. Besides the aforementioned Gibberellins (GA_1_, GA_3_, and GA_4_) and 6-Benzyladenine (BAP), this cluster also enriched various cytokinin substances, including Kinetin (K), N6-Isopentenyl-adenine-9-glucoside (iP9G), and para-Topolin riboside (pTR). Compared with other stages, these metabolites’ expression levels were significantly altered after hormone treatment (Figure 8B), suggesting an important role in hormone-mediated regulation of flower bud differentiation. Further quality control analysis revealed that the expression distribution (box plot) and density curve of these DAMs were highly consistent (Figure 8C,D), not only verifying the reproducibility of the experimental data but also confirming the reliability of the conclusions. This discovery provides new metabolomic insights into the molecular mechanism of exogenous hormone regulation of flower bud differentiation.

### 2.8. Combined Analysis of Transcriptomics and Metabolomics

**Figure 9:**
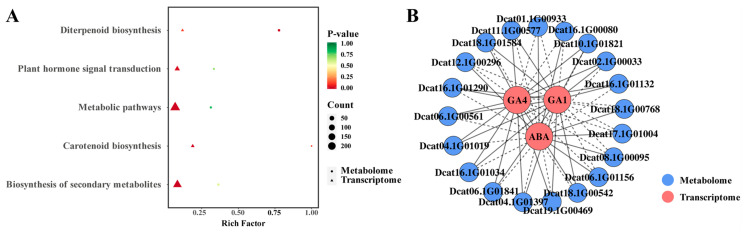
Common KEGG analysis (**A**). Correlation analysis of DEGs and DAMs in plant hormone signal transduction pathways (**B**). Note: The solid line represents a positive correlation, while the dotted line represents a negative correlation.

Integrated analysis of transcriptomic and metabolomic data revealed a convergence: many DEGs and DAMs were significantly enriched in the plant hormone signal transduction pathway (Figure 9A). This robust enrichment underscores the pivotal role of this pathway in orchestrating the hormone-promoted flower bud differentiation process in *D. officinale.*

Further elucidating the regulatory interplay within this pathway, correlation network analysis (Figure 9B) revealed intricate connections between key DEGs and DAMs in the D3 vs. D2 comparison. Notably, we observed strong positive correlations between specific genes (e.g., Dcat06.1G01841) and the upregulated hormones GA1 and GA4. Conversely, genes associated with ABA signaling (e.g., Dcat18.1G01584, Dcat16.1G01290) showed negative correlations with ABA. This gene–metabolite correlation network provides compelling evidence for the coordinated action of exogenous 6-BA and GA_3_ in modulating the expression of hormone-responsive genes and the accumulation of key signaling molecules, thereby synergistically driving flower bud differentiation.

### 2.9. Responses of Plant Hormone Pathways to Hormones

**Figure 10:**
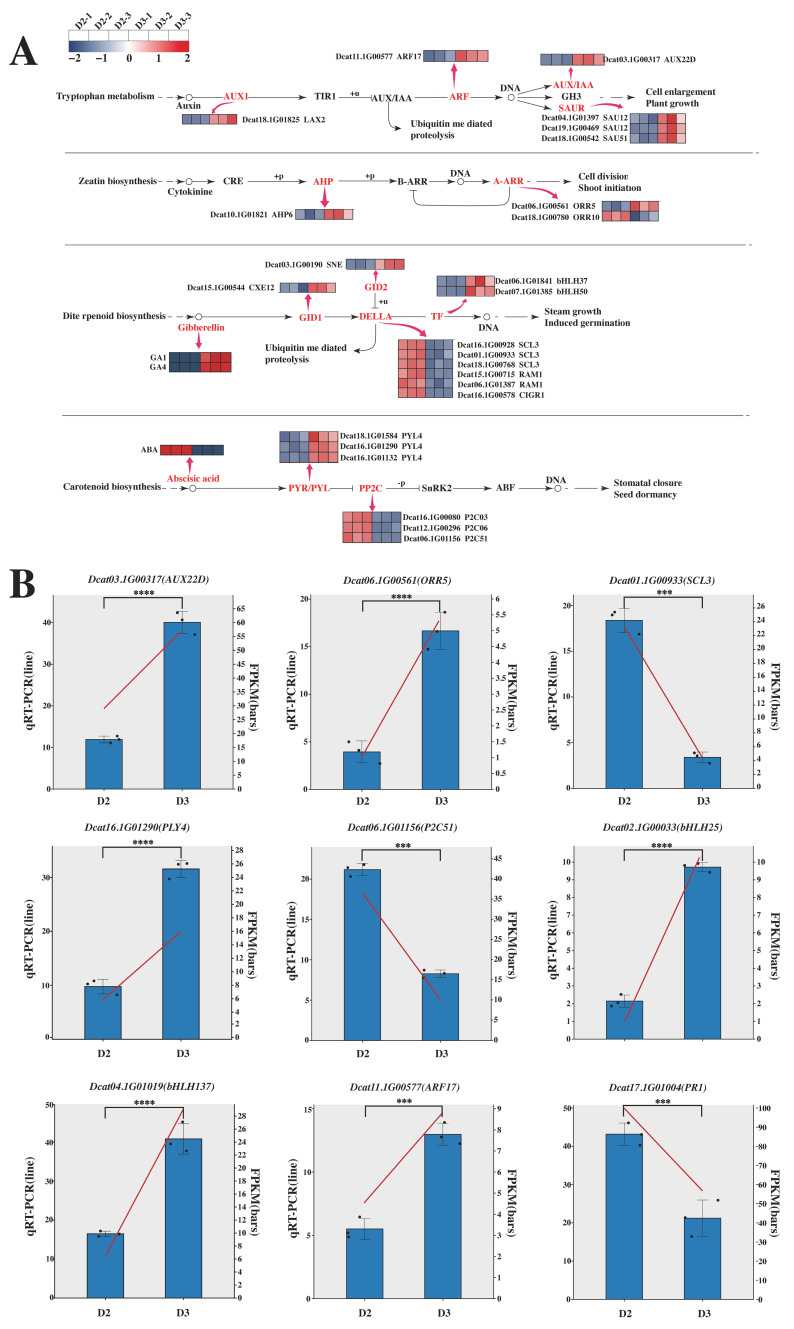
Annotation of DEGs and metabolites to plant hormone signal transduction pathways (**A**). qRT-PCR verification results of DEGs related to hormone treatment (**B**). Note: *** *p* < 0.001; **** *p* < 0.0001, The three dots on the pillar represent three repetitions.

The above findings reveal a direct molecular connection between the gene expression changes (identified by transcriptomics) and metabolic alterations (detected by metabolomics) triggered by the combined hormone treatment. To delve deeper into the specific responses of these interconnected hormone pathways to the exogenous 6-BA and GA_3_ treatment, we analyzed the expression patterns of key pathway components. In this study, we identified 25 DEGs in the comparisons of D3 vs. D2. These DEGs were mainly concentrated in the auxin, cytokinin, gibberellin, and abscisic acid pathways. Among the 25 DEGs involved in these pathways, one *AUX1* (Dcat18.1G01825), one *AUX/IAA* (Dcat03.1G00317), one *ARF* (Dcat11.1G00577), and three *SUARs* (Dcat19.1G00469, Dcat18.1G00542, and Dcat04.1G01397) were involved in the auxin signaling pathway, and all of these genes were upregulated after hormone treatment; one *AHP* (Dcat10.1G01821) and two *A-ARRs* (Dcat06.1G00561 and Dcat18.1G00780) were differentially expressed in the comparison of D3 vs. D2 in the cytokinin pathway; one *GID1* (Dcat15.1G00544), one *GID2* (Dcat03.1G00190), six *DELLA* (Dcat18.1G00768, Dcat01.1G00933, Dcat16.1G00928, Dcat16.1G00578, Dcat06.1G01387, and Dcat15.1G00715), and two TFs (Dcat04.1G01019 and Dcat06.1G01841) were upregulated in the gibberellin pathway; and three *PYR/PYL* (Dcat16.1G01290, Dcat16.1G01132, and Dcat18.1G01584) were upregulated and three *PP2Cs* (Dcat16.1G00080, Dcat12.1G00296, and Dcat06.1G01156) were downregulated in the comparison of D3 vs. D2 in the abscisic acid pathway. Additionally, GA_1_ and GA_4_ in the gibberellin pathway were upregulated after hormone treatment, while ABA in the abscisic acid pathway was downregulated (Figure 10A).

### 2.10. Real-Time Fluorescence Quantitative PCR (qPCR) Analysis

Given that the combined exogenous application of 6-BA and GA_3_ promotes flower bud differentiation in D. officinale, likely by altering endogenous hormone levels, we verified selected gene expression by real-time quantitative RT-PCR. We selected one representative gene from each of the auxin, cytokinin, GA, and ABA pathways, and additionally chose the five genes most highly correlated with levels of ABA, GA_1_, and GA_4_ for verification. After hormone treatment, the expression trends of these genes in *D. officinale* flower buds were consistent with the transcriptome data, despite some differences in expression levels. This indicates that the RNA-Seq data obtained in this study are highly reliable (Figure 10B).

## 3. Discussion

In China, nearly 80 species of the genus *Dendrobium* have been identified [25,26]; among them, 33 species serve as source plants for commercial Dendrobium cultivation [27]. D. officinale, one of the five Dendrobium species listed in the Chinese Pharmacopoeia, is found in many countries (for example, Japan, the United States, and Australia) and is widely distributed in China [28]. As a traditional medicinal plant, the stem of *D. officinale* has become the main focus of modern research and industry due to its richness in active compounds such as polysaccharides, alkaloids, and various amino acids. The stems are usually processed by boiling into a product called ‘Tie Pi Congee’ (Iron Skin Congee) or by segmented drying. Such processing methods not only effectively extend the storage period but also significantly enhance its commercial value as an herbal medicine [29]. By contrast, comprehensive utilization of *D. officinale* flowers has not yet been fully developed. Studies have shown that *D. officinale* flowers have both ornamental and medicinal value; for example, flower tea exhibits anti-cancer effects [30]. It is worth noting that the efficiency of flower bud differentiation directly affects the reproductive cycle and synchrony of flowering in D. officinale. The plant must accumulate sufficient nutrients to transition from vegetative to reproductive growth. At the same time, the accumulation of carbohydrates is closely related to flower bud differentiation [31]. It has been reported that the combined treatment of GA_3_ and 6-BA can promote flower bud differentiation and increase carbohydrate content [16,32]. Based on this, we hypothesized that these two hormones could promote flower bud differentiation in *D. officinale*, and we tested their effects accordingly. Experimental data showed that different hormone treatments significantly promoted flower bud differentiation in D. officinale. Among them, T3 (200 ppm 6-BA + 50 ppm GA_3_) produced the most flowers. However, T2 (50 ppm GA3) caused leaf yellowing and abscission, possibly due to an imbalance in endogenous hormones and insufficient nutrient supply [33]. The physiological measurements showed that soluble protein and sugar content in T3 peaked at stage II, and starch content gradually increased during differentiation, remaining higher than in other treatments throughout the process. Starch, the primary form of carbohydrate storage in plants, is continuously consumed during flower bud differentiation to maintain normal metabolism. Thus, high starch levels are beneficial for flower bud differentiation [34]. Studies have shown that during the flower bud differentiation process of plants, peroxidase (POD) and catalase (CAT), as important antioxidant enzymes, play a crucial role in initiating and completing the flower bud differentiation by maintaining the balance of oxygen free radicals within the cells [35,36,37]. In our study, POD and CAT activities in the hormone-treated groups were lower than in the control at stage I, then gradually increased and peaked at stage III. Interestingly, T3 treatment induced a smaller increase in POD activity than single-hormone treatments, but it produced a significantly larger late-stage increase in CAT activity. This indicates that the two hormones have complementary effects on the antioxidant system: 6-BA may maintain energy supply by regulating carbon metabolism, whereas GA_3_ enhances antioxidant enzyme synthesis via gene expression regulation. The synergistic effect of the two hormones is more crucial in the later stage of differentiation. The above data indicate that the T3 treatment method has practical significance for promoting the flower bud differentiation of *D. officinale* and increasing the number of flower buds.

We further performed transcriptome and metabolome analyses to identify key genes and metabolites responding to exogenous GA_3_ and 6-BA. A total of 12,500 DEGs and 113 DAMs were identified across five stages; among these, 1713 DEGs and 18 DAMs were associated with hormone treatment (stage D3). Based on the analysis of metabolic group differences, 14 of the 18 DAMs showed upregulation after hormone treatment, including seven cytokinins (para-Topolin, para-Topolin riboside, 2-methylthio cis zeatin, N6-Isopentenyl-adenine-9-glucoside, 6-Benzyladenosine, 6-Benzyladenine, Kinetin), six gibberellins (Gibberellin A4, Gibberellin A5, Gibberellin A1, Gibberellin A3, Gibberellin A20, Gibberellin A53), and one jasmonic acid (12-Oxophytodienoic acid). One gibberellin (Gibberellin A8), one jasmonic acid (Jasmonate-1-aminocyclopropane-1-carboxylic acid), one Abscisic acid (ABA), and one strigolactone (Strigol) showed decreased content after GA_3_ and 6-BA treatment. Therefore, the application of exogenous 6-BA and GA_3_ will disrupt the endogenous hormone balance. Studies have shown that apple (Malus domestica) cultivars with different flowering capacities exhibit significant differences in endogenous cytokinin (CTK), abscisic acid (ABA), and gibberellin (GA) levels [38]. During the flowering induction stage, auxin (IAA) and abscisic acid (ABA) levels increase, while gibberellin (GA) levels decrease. This further confirms the role of hormonal networks in regulating the flowering process [39]. Additionally, exogenous salicylic acid (SA) treatment can affect flowering by promoting auxin accumulation and inhibiting gibberellin biosynthesis and signaling [40]. These metabolites may provide insights into the molecular mechanisms of hormone regulation of orchid flower bud differentiation.

Transcription factors play a crucial role in the growth and development of plants. *C2H2* zinc finger proteins have the largest number of zinc-finger domains and are ubiquitous in eukaryotic cells [41,42]. Studies have shown that *C2H2-ZFPs* play an important role in the transcriptional regulation of flower development, especially in the development of flower organs and pollen formation [43,44]. WGCNA has been widely used in the research into multiple species to analyze the association between gene expression patterns and physiological traits [45,46]. Through WGCNA, we identified 1130 hormone-related genes and found that *C2H2* (Dcat18.1G0098) acted as a core hub transcription factor. *C2H2* was significantly correlated with 35 genes, including members of the *AP2/ERF*, *ARF*, and *C2C2-Dof* families, all of which were upregulated after hormone treatment. The expression of these transcription factors was significantly upregulated after hormone treatment. The *AP2/ERF* transcription factor family regulates multiple key processes in plants, including growth and development, fruit ripening, flower formation, and stress response. In *kiwifruit*, *AeAP2/ERF061* and *AeAP2/ERF067* are highly expressed in floral organs. In *dragon* fruit, Dlo_015581.1, an *AP2/ERF* family gene highly expressed in flower buds, is homologous to *Arabidopsis* AT2G28550.1 (*AtRAP2.7*) [47]. At the same time, in *Arabidopsis*, *RAP2.7* has been identified as an *AP2* gene that promotes flower development [48]. Additionally, the *ARF* and *Dof* transcription factor families have been confirmed to be closely related to flower bud differentiation and flower development [49,50]. In conclusion, *C2H2* (Dcat18.1G0098) acts as a key hub gene, forming a synergistic regulatory module with *AP2/ERF*, *ARF*, and *Dof* family transcription factors to drive hormone-mediated flower development.

Plant hormones, as endogenous regulatory molecules, span all developmental stages of plants, from embryogenesis to reproductive maturity, playing a core regulatory role in the plant life cycle. Studies on model plants have shown that different hormone signaling pathways form complex regulatory systems through dynamic interaction networks, with both antagonistic and synergistic effects [51]. In this study, we observed significant changes in the DEGs and DAMs enriched in the plant hormone signal transduction pathway, indicating that they play an important regulatory role in the flower bud differentiation process of *D. officinale*. The auxin signaling pathway is the main mechanism by which plants respond to auxins such as indole-3-acetic acid (IAA). Auxin directly binds to TIR1/AFB receptor proteins in the nucleus, increasing the receptor’s affinity for AUX/IAA repressor proteins. This triggers ubiquitination and the subsequent degradation of AUX/IAA by the 26S proteasome. The degradation of AUX/IAA releases the repressed ARF transcription factors, allowing ARFs to bind to auxin response elements in target gene promoters and activate or inhibit downstream gene expression, thereby promoting cell expansion and plant growth [52,53]. In this study, *AUX1* (Dcat18.1G01825), an auxin influx carrier gene, was upregulated. This is consistent with the observation that combined 6-BA and GA_3_ treatment upregulated *ARF* (Dcat11.1G00577), *AUX/IAA* (Dcat03.1G00317), and *SAUR* (Dcat04.1G01397, Dcat19.1G00469, and Dcat18.1G00542) genes. This indicates that in *D. officinale*, the differentiation of flower buds requires a higher level of *AUX* and signal transduction, and the use of hormones promotes this process. Cytokinin binds to histidine kinase receptors on the plasma membrane (e.g., *AHK2/3/4*), triggering receptor autophosphorylation. The phosphate group is then transferred via an *AHP* phosphotransfer protein to a B-type response regulator (*B-ARR*) in the nucleus. Activated *B-ARR* regulates downstream genes, repressing senescence-related genes and activating cell division genes, thereby driving cell proliferation and bud primordium development. This plays a decisive role in the initiation of flower bud differentiation [54]. In our results, hormone treatment increased cytokinin levels and upregulated related signaling genes (e.g., *AHP* gene Dcat10.1G01821 and *A-ARR* gene Dcat06.1G00561), while downregulating another *A-ARR* gene (Dcat18.1G00780). Studies have shown that *AHP6* is a target of the *DRNL* transcription factor, and the loss of *DRNL* function would significantly affect the development of floral organs [55]. Gibberellin acts as a signaling molecule and binds to the *GID1* receptor, promoting the degradation of *DELLA* negative regulatory proteins, thereby relieving the inhibition of *PIF* and other transcription factors and ultimately promoting physiological processes such as stem elongation, seed germination, and flower bud differentiation [56]. In the results of this study, GA_1_ and GA_4_ were upregulated as active forms of gibberellin, indicating that the biosynthesis of gibberellin was significantly enhanced after hormone treatment. This is consistent with the upregulation of *GID1* (Dcat15.1G00544), *GID2* (Dcat03.1G00190), and transcription factor (Dcat06.1G01841 and Dcat07.1G01385) genes by hormone treatment, and the downregulation of *DELLA* (Dcat16.1G00928, Dcat01.1G00933, Dcat18.1G00768, Dcat15.1G00715, Dcat06.1G01387, and Dcat16.1G00578) genes. Notably, the combined application of 6-BA and GA_3_ upregulated *bHLH* transcription factors, which play important roles in regulating flower bud differentiation and flower development [57]. Carotenoids are precursors for ABA synthesis. After ABA binds to the receptor *PYR/PYL*, it inhibits the activity of *PP2C* phosphatase, thereby activating *SnRK2* kinase. Activated *SnRK2* then phosphorylates downstream target proteins, such as transcription factor *ABF*, regulating the expression of seed dormancy genes [58]. In our results, ABA levels were lower in the hormone-treated group compared to the control. This was accompanied by upregulation of *PYR/PYL* genes (Dcat18.1G01584, Dcat16.1G01290, and Dcat16.1G01132) and downregulation of *PP2C* genes (Dcat16.1G00080, Dcat12.1G00296, and Dcat06.1G01156) in the hormone-treated plants. These results suggest that high ABA levels may inhibit flower bud differentiation in *D. officinale.* The observed decrease in ABA and reduced ABA signaling after hormone treatment imply that exogenous 6-BA and GA_3_ can synergistically promote flower bud differentiation by lowering endogenous ABA levels and suppressing ABA signaling.

While our multi-omics approach provides comprehensive insights into hormone-regulated flower bud differentiation in *D. officinale*, certain limitations warrant acknowledgment. Firstly, the functional roles of candidate regulators identified through WGCNA remain experimentally unvalidated. Future studies employing gene silencing or overexpression approaches are needed to confirm their causal relationships with floral transition. Secondly, the generalizability of this molecular framework to other orchid species requires verification. Comparative analyses across diverse orchids would elucidate conserved versus species-specific regulatory mechanisms. Such validations would strengthen the translational potential of these findings for precision breeding in Orchidaceae.

## 4. Materials and Methods

### 4.1. Plant Materials and Experimental Design

*D. officinale* seedlings were commercially sourced. All two-year-old seedlings exhibited uniform vigor under standardized cultivation (18–28 °C, natural light, 70–85% humidity), showing optimal growth without disease. We established three treatments: T1 (200 ppm 6-BA), T2 (50 ppm GA_3_), T3 (200 ppm 6-BA + 50 ppm GA_3_), with a distilled water control (CK). Initial treatment commenced on 7 March 2024 during the vegetative phase, concluding on 6 May 2024 with post-floral morphogenesis. The first treatment was carried out on 7 March 2024 (at this time, the plant was still in the vegetative growth stage), and the treatment ended on 6 May 2024 (by then, the flower bud morphological differentiation was completed). During this period, the treatment was conducted once every 30 days. Leaf samples were collected starting from the second treatment, once every 30 days, for a total of three times, which were marked as I, II, and III (these samples were used for physiological parameters determination), and the cell and tissue morphology of the flower buds was observed using paraffin sectioning technology [59]. Scanning electron microscopy (Thermo Fisher Biotechnology Co., Ltd., Guangzhou, China) delineated five floral developmental stages: D1–D5, where D3 represents a combined 50 ppm GA_3_ + 200 ppm 6-BA treatment. Initial sampling in early March (vegetative phase) captured floral bud morphological variation. Hormone application occurred during mid-flower bud differentiation (mid-April), with final sampling on 6 May 2024 following morphogenesis completion. Each treatment included triplicate biological replicates for physiological, transcriptomic, and metabolomic analyses. Three independent biological replicates per group were processed separately for omics analyses. For transcriptomics and metabolomics, buds from 3 plants were pooled per replicate (fresh weight ≥ 3.6 g) to capture biological variation. All samples were frozen in liquid nitrogen and stored at −80 °C for subsequent use.

### 4.2. Physiological Parameters Determination

We quantified soluble sugars, proteins, starch, peroxidase (POD), and catalase (CAT) activities in *D. officinale.*

#### 4.2.1. Determination of Soluble Sugar and Starch

Soluble sugar and starch contents were determined using the anthrone–sulfuric acid method [60]. Approximately 0.1 g of seed powder was thoroughly mixed with 10 mL of 80% ethanol in a centrifuge tube, boiled in an 80 °C water bath for 30 min, and centrifuged; the supernatant and precipitate were used for soluble sugar and starch analysis, respectively. For soluble sugars, 2 mL of supernatant was transferred to a new tube, mixed with 0.5 mL anthrone–ethyl acetate solution (1 g anthrone dissolved in 50 mL ethyl acetate) and 5 mL concentrated sulfuric acid, boiled in a 100 °C water bath for 1 min, and cooled to room temperature, and absorbance was measured at 630 nm using a spectrophotometer. For starch, the precipitate was transferred to a 50 mL volumetric flask, mixed with 20 mL distilled water, boiled in a 100 °C water bath for 30 min, treated with 2 mL of 9.2 mol/L perchloric acid for 15 min, cooled to room temperature, and centrifuged, and the supernatant was analyzed using the anthrone method described above.

#### 4.2.2. Determination of Soluble Protein

Protein content was determined using the Coomassie brilliant blue method [61]. Approximately 0.02–0.05 g of seed powder was homogenized with distilled water, centrifuged at 4000× *g* for 10 min, and 1 mL of the supernatant was transferred to a new centrifuge tube. Then, 5 mL of Coomassie brilliant blue reagent (0.1 g Coomassie brilliant blue dissolved in 50 mL 90% ethanol, mixed with 100 mL 85% (*w*/*v*) phosphoric acid, and diluted to 1 L with distilled water) was added. Absorbance was measured at 595 nm using a spectrophotometer.

#### 4.2.3. Determination of POD Activity

Frozen samples from different stages and NO treatments were pulverized under liquid nitrogen using an IKA^®^ A11 basic analytical mill (IKA^®^, Staufen, Germany). The resulting powder was homogenized in 100 mM of Tris-HCl buffer (pH 8.0) containing 0.1% (*v*/*v*) Triton X-100, 1 mM ethylenediaminetetraacetic acid (EDTA), and 10% (*v*/*v*) glycerol, at a final ratio of 1:1 (*w*/*v*, plant material/buffer). The homogenate was centrifuged at 15,000× *g* for 30 min at 4 °C. The supernatant was collected and used for the enzymatic peroxidase (POD) activity assay. The protein concentration of the samples was determined using the Bio-Rad protein assay (Hercules, CA, USA), with bovine serum albumin (BSA) as the standard [62].

#### 4.2.4. Determination of CAT Activity

After grinding the sample into a fine powder with liquid nitrogen, accurately weigh 0.1 g of the sample and add 0.5 mL of PBS buffer. Homogenize the mixture in an ice bath, then centrifuge the homogenate at 8000× *g* for 10 min at 4 °C. Collect the supernatant for subsequent analysis. Pipette 20 μL of the supernatant into a 1.5 mL Eppendorf tube, add 100 μL of 20 μmol/mL hydrogen peroxide (H_2_O_2_) standard solution, and mix thoroughly. Incubate the reaction mixture in a water bath at 25 °C for 10 min. Then, add 180 μL of 50 mmol/L ammonium molybdate tetrahydrate solution and mix well. Let the mixture stand at room temperature for 10 min. Transfer 200 μL of the reaction solution to a 96-well microplate and measure the absorbance at 405 nm. For the control group, replace the 20 μL of H_2_O_2_ standard solution with 20 μL of distilled water. For the standard group, replace the 20 μL of sample supernatant with 20 μL of PBS buffer. For the blank group, replace both the 20 μL of sample supernatant and the 20 μL of H_2_O_2_ standard solution with 20 μL of PBS buffer and 20 μL of distilled water, respectively [63].

The determination of nutritional components was performed at the Wuhan ProNets Testing Technology Co., Ltd. (Wuhan, China). Data were analyzed using SPSS 26.0 with Duncan’s multiple range test for significance (*p* < 0.05).

### 4.3. cDNA Library Construction, Sequencing, and Data Analysis

Flower bud samples from five developmental stages were flash-frozen on dry ice and commercially sequenced (Illumina NovaSeq 6000, Illumina, San Diego, CA, USA) by a biotech service provider (Wuhan, China), including library construction and quality control. Samples were extracted by ethanol precipitation and CTAB-PBIOZOL. After successful extraction, RNA was dissolved by adding 50 µL of DEPC-treated water. Subsequently, total RNA was identified and quantified using a Qubit fluorescence quantifier and a Qsep400 high-throughput biofragment analyzer. Post-sequencing, raw reads underwent quality filtering: adapter removal and exclusion of low-quality bases (Q < 20) generated clean reads. Clean reads were aligned to the *Dendrobium* reference genome using HISAT2 with default parameters, generating mapped reads. All unique genes were compared with Kyoto Encyclopedia of Genes and Genomes (KEGG), Gene Ontology (GO), Non-redundant Protein Database (NR), Swiss-Prot, TrEMBL, and EuKaryotic Orthologous Groups (KOG) using diamond, with E-value ≤ 1 × 10^−5^.

### 4.4. Identification and Analysis of DEGs

To accurately reflect the transcriptional expression levels of all samples, we used fragments per kilobase of transcript per million fragments mapped (FPKM) as the indicator to measure the expression levels of transcripts or genes. Subsequently, we conducted differential expression analysis between samples using DESeq2 and performed multiple hypothesis testing correction on the *p*-value using the Benjamini–Hochberg method. DEGs were defined by |log_2_FC| ≥ 1 and adjusted *p*-value < 0.05. Based on the k-means clustering method, we clustered the DEGs at different stages [64]. KEGG pathway and gene ontology (GO) enrichment analyses were performed on DEGs using the Metware Cloud, a free online platform for data analysis (https://cloud.metware.cn), with results visualized in heatmaps. Enrichment analysis was performed based on the hypergeometric test, with pathway-based hypergeometric distribution testing for KEGG and GO term-based analysis for GO. In the KEGG pathway analysis, the formula for calculating enriched *p*-values was

$$P=1-\sum_{i=0}^{m-1}\frac{\left(\frac{M}{i}\right)\left(\frac{N-M}{n-i}\right)}{\left(\frac{N}{n}\right)}$$

here, *N* is the number of all genes that have a KEGG annotation, n is the number of DEGs in *N*, *M* is the number of all genes annotated to specific pathways, and *m* is the number of DEGs in *M*. A *p*-value of 0.05 was selected as the threshold for deciding whether a gene set was significantly enriched.

### 4.5. Quantitative Real-Time Polymerase Chain Reaction Verification

For the qRT-PCR verification, nine genes randomly selected from the DEGs related to hormones were used. The primer sequence information is presented in Table S2. RNA was extracted, and cDNA was generated using the reverse transcription kit provided by Wuhan Maiwei Metabolic Biotechnology Co., Ltd (Wuhan, China). Specific primers for the required genes were designed using Primer 6.0 software, and their specificity was verified through the NCBI database. Actin was used as the internal reference. qRT-PCR was performed using 2X SYBR Green Fast qPCR Mix (Biomarker, Beijing, China) on a CFX96 real-time PCR system (Bio-Rad, Hercules, CA, USA). A 20 μL reaction system was used, with the following conditions: 95 °C for 30 s, 95 °C for 5 s, 60 °C for 30 s, and 72 °C for 30 s, for a total of 40 cycles. The quantitative data obtained were calculated for relative expression using the 2^−ΔΔCt^ method [65].

### 4.6. Metabolite Analysis

The hormone contents in 15 flower bud samples were analyzed using the AB Sciex QTRAP 6500 LC-MS/MS platform in conjunction with MetWare (http://www.metware.cn/). A total of 50 mg of plant sample was weighed into a 2 mL plastic microtube, frozen in liquid nitrogen, and dissolved in 1 mL methanol/water/formic acid (15:4:1, *v*/*v*/*v*). A total of 10 μL internal standard mixed solution (100 ng/mL) was added to the extract as internal standards (IS) for the quantification. The mixture was vortexed for 10 min, then centrifuged for 5 min (12,000× *g*/min, and 4 °C), after which the supernatant was transferred to clean plastic microtubes; this was followed by evaporation to dryness, dissolution in 100 μL 80% methanol (*v*/*v*), and filtering through a 0.22 μm membrane filter for further LC-MS/MS analysis [66,67]. The sample extracts were analyzed using an UPLC-ESI-MS/MS system (UPLC, ExionLC™ AD, https://sciex.com.cn/; MS, QTRAP^®^ 6500+, https://sciex.com.cn/). The analytical conditions were as follows, LC: column, Waters ACQUITY UPLC HSS T3 C18 (100 mm×2.1 mm i.d, 1.8 µm); solvent system, water with 0.04% acetic acid (A), acetonitrile with 0.04% acetic acid (B); gradient program, started at 5% B (0–1 min), increased to 95% B (1–8 min), 95% B (8–9 min), finally ramped back to 5% B (9.1–12 min); flow rate, 0.35 mL/min; temperature, 40 °C; injection volume, 2 μL [68,69,70]. Linear ion trap (LIT) and triple quadrupole (QQQ) scans were acquired on a triple quadrupole-linear ion trap mass spectrometer (QTRAP), QTRAP^®^ 6500+ LC-MS/MS System, equipped with an ESI Turbo Ion-Spray interface, operating in both positive and negative ion mode and controlled by Analyst 1.6.3 software (Sciex). The ESI source operation parameters were as follows: ion source, ESI+/−; source temperature, 550 °C; ion spray voltage (IS), 5500 V (positive), −4500 V (negative); and the curtain gas (CUR) was set at 35 psi. Phytohormones were analyzed using scheduled multiple reaction monitoring (MRM). Data acquisitions were performed using Analyst 1.6.3 software (Sciex). Multiquant 3.0.3 software (Sciex) was used to quantify all metabolites. Mass spectrometer parameters, including the declustering potentials (DP) and collision energies (CE) for individual MRM transitions, were performed with further DP and CE optimization. A specific set of MRM transitions was monitored for each period according to the metabolites eluted within this period [71,72,73].

The HCA (hierarchical cluster analysis) results of samples and metabolites were presented as heatmaps with dendrograms. HCA was carried out by R package (version 1.2.1) pheatmap. For HCA, normalized signal intensities of metabolites (unit variance scaling) are visualized as a color spectrum. Significantly regulated metabolites between groups were determined by absolute Log2FC (fold change). Identified metabolites were annotated using the KEGG compound database (http://www.kegg.jp/kegg/compound/ (accessed on 4 June 2025); annotated metabolites were then mapped to the KEGG Pathway database (http://www.kegg.jp/kegg/pathway.html (accessed on 4 June 2025). Pathways with significantly regulated metabolites mapped to were then fed into MSEA (metabolite sets enrichment analysis), and their significance was determined by the hypergeometric test’s *p*-values.

### 4.7. Gene Network Construction and Visualization

To investigate the regulatory mechanism of hormones controlling flower bud differentiation in *D. officinale* and potential transcription factors, we constructed a coexpression network using the WGCNA package in R with the following parameters: powerEstimate = 18, minModuleSize = 50, and mergeCutHeight = 0.25 [74]. The dynamic tree cutting method divided genes into coexpression modules, where modules with similar clustering were merged. Intra-module gene correlations (module membership, MM) and inter-module relationships were quantified, enabling selection of key modules associated with hormonal regulation for downstream analysis. Network visualization was performed with Cytoscape v3.10.0.

### 4.8. Combined Transcriptome and Metabolome Analysis

By using the cor function in R to calculate the Pearson correlation coefficient between genes and metabolites, a correlation analysis was conducted for all quantitative values in the samples. The absolute value of the Pearson correlation coefficient |PCC| was greater than 0.8, and the *p*-value was less than 0.05. The diagrams were created using Adobe Illustrator 2024, and the DEGs and differentially expressed metabolites were annotated to the relevant KEGG pathways.

## 5. Conclusions

This study systematically analyzed the molecular mechanisms by which 6-BA and GA_3_ coordinately regulate flower bud differentiation in *D. officinale*, integrating physiological measurements, transcriptomics, metabolomics, and WGCNA analysis. The experiment confirmed that the combination treatment of 200 ppm 6-BA and 50 ppm GA_3_ (T3) significantly promoted flower bud differentiation, increased the number of flowers, and induced fluctuations in soluble sugar, protein, and starch content across these stages, providing a material basis for flower bud development. Transcriptome analysis identified 11,994 DEGs, among which DEGs in the hormone combined treatment stage (D3) were significantly enriched in plant hormone signal transduction and plant–pathogen interaction pathways. Metabolomics further revealed that 18 DAMs (such as GA_3_, BAP, and OPDA) were specifically enriched in the D3 stage, and their accumulation dynamics were highly correlated with the flower bud differentiation process. Through WGCNA, the hub genes of the “yellow module” (including *C2H2*, *bZIP*, and *NAC* family transcription factors) were screened out, and their interaction network significantly regulated the expression of hormone response genes. In summary, this study constructs, for the first time, a molecular framework of flower bud differentiation in *Dendrobium* based on the metabolomic–transcriptomic coordination network driven by 6-BA and GA_3_. Key hub genes (such as *C2H2*) form regulatory modules by interacting with transcription factors such as *AP2/ERF* and *ARF*, while the dynamic changes of endogenous hormones (such as the increase in the active form of GA and the decrease in ABA levels) further strengthen the flowering signal. This achievement provides key targets for the precise regulation of flowering time and molecular breeding in orchid plants.

## Figures and Tables

**Table 1:** Effects of different hormone treatments on flower bud differentiation.

Species	Hormone Concentration (ppm)	Effect	Reference
	**6BA**		
*Mango*	200	Promotes early flowering	[15]
*Gossypium hirsutum* L.	25	Increases the number of flower buds	[16]
*Phalaenopsis*	150	Promotes the formation and development of flower stems	[17]
	**GA_3_**		
*Limonium* spp. *‘Ocean Blue’*	400	Promotes early flowering	[18]
*Paeonia suffruticosa Andr*	800/900	Promotes early flowering	[19]
*Lilium x elegans cvs. Fangio*	50	Improves the quality of flowers; promotes early flowering	[20]
*Dianthus caryophyllus*	400	Improves the quality of flowering; promotes early flowering	[21]
	**6BA + GA_3_**		
*Dendrboium Nobile*	200 + 50	Promotes flower bud differentiation	[22]
*Dendrobium ‘White Christmas’*	100 + 50	Promotes early flowering; increases the number of flowers	[23]
*Cymbidium sinense*	100 + 50	Promotes flower bud differentiation; increases the number of flower buds; promotes early flowering	[24]

## Data Availability

This study did not use publicly available data; the transcriptome and metabolome data were provided by Metware Biotechnology Co., Ltd.

## Supplementary Materials

The following supporting information can be downloaded at: https://www.mdpi.com/article/10.3390/plants14172668/s1, Figure S1: Analysis of the number of upregulated and downregulated DEGs in D2 vs. D1, D4 vs. D2, and D5 vs. D4.; Figure S2: DEG enrichment analysis for D2 vs. D1, D4 vs. D2, and D5 vs. D4; Table S1: Statistical table of sequencing data quality output; Table S2: Primer information; Table S3: The components of the internal standard mixture solution.
