# Supplementary material for: Regulation of Flower Bud Differentiation Hormones and Identification of Related Key Genes in Dendrobium officinale Based on Multi-omics Analysis
Source: Plants (Basel). 2025 Aug 27;14(17):2668. doi: 10.3390/plants14172668 (PMC12430784; doi:10.3390/plants14172668)
Supplement: Supplementary file 1 [file plants-14-02668-s001.zip › plants-3767479-supplementary.pdf]

Supplementary Table 1. Statistical table of sequencing data quality output.

Sample	Clean Base (G)	Q20 (%)	Q30 (%)	GC Content (%)
D1-1	8.13	98.97	96.68	45.60
D1-2	7.53	98.96	96.64	46.59
D1-3	8.31	99.06	96.94	46.44
D2-1	7.05	97.99	93.98	46.38
D2-2	8.51	98.82	96.21	46.23
D2-3	8.13	98.83	96.24	46.34
D3-1	8.09	99.03	96.86	46.11
D3-2	7.65	98.98	96.71	46.14
D3-3	9.54	98.84	96.24	45.99
D4-1	7.35	98.7	95.83	46.51
D4-2	7.38	98.93	96.52	46.55
D4-3	9.24	98.79	96.11	46.48
D5-1	7.50	99.01	96.77	46.48
D5-2	7.44	98.89	96.38	46.69
D5-3	8.09	99.00	96.78	46.45

Note: Q20%: the percentage of bases with a Qphred value of no less than 20 among the total bases; Q30%: the percentage of bases with a Qphred value of no less than 30 among the total bases; GC Content %: the percentage of the sum of G and C bases among the total bases in high-quality reads.

Supplementary Table 2. Primer information.

Gene ID	Forward primer (5' to 3')	Reverse primer (5' to 3')
AUX22D	ATGGCAACTGAAATGGAAATT	TTATTTTTCAGACATCCCCTT
ORR5	ACCGAATGTGAGCATGATTATCA	CCTTGACCTTCTTCAGGAGTTCATA
SCL3	CCTGGCGTGGCTTCTACATC	AGGGCCTTCTTTGCGGTCT
PYL4	CCGTCGTCCAGCAAATCG	ACATCTCCATCGCCAACAAC
P2C51	TGGTGACGTGCTGTTCACAT	AATCATACCTCCTGCTGCCG
bHLH25	GCTCCTCTAGTTCCCATCAAAGA	TATGACGAAATTCTAACCCGTAC
bHLH137	AAGGTCACTGGAAAAGCCCTC	CCATCCCTTCGATCTTCTGAGTTA
ARF17	TTAGTGGCAGTCAGGATG	GCAGTTGAGGTTGAGTTG
PR1	ATGTCAAAGTACACCATT	TCAATAAGGCCTCTCTCC
Actin	CCCTACCTCCTACCTCTGCG	GCAAACCCAGCCTTCACCAT

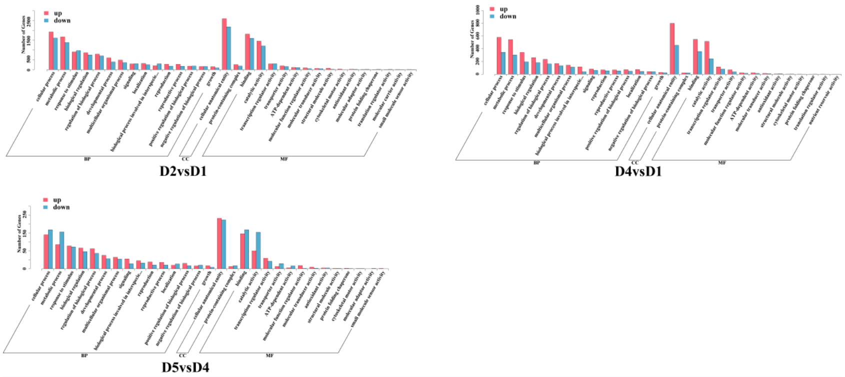

Figure S1. Analysis of the number of upregulated and downregulated DEGs in D2 vs. D1, D4 vs. D2, and D5 vs. D4.

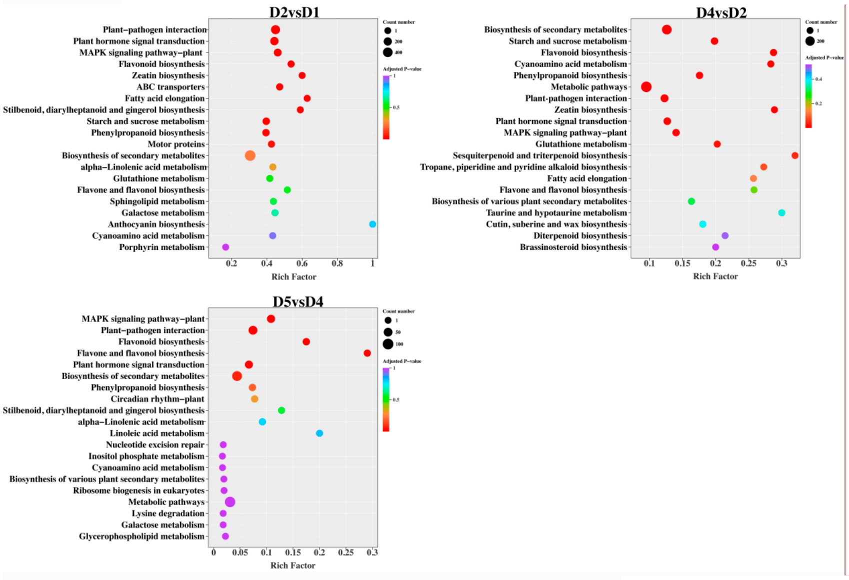

Figure S2. DEG enrichment analysis for D2 vs. D1, D4 vs. D2, and D5 vs. D4.

Commented [1]: Attention AE: replace hyphens with en dashes and add Oxford commas where needed.

Supplementary Table 3. The components of the internal standard mixture solution.

	Number	Ion ID	Name	Specification
**Internal Standard**	1	IS_ABA	d6-ABA	1 mg
	2	IS_IAA-Val	d5-IAA-Val	50ug
	3	IS_IBA	d2-IBA	100mg
	4	IS_IAA	d2-IAA	100mg
	5	IS_ICA	Indole-3-carboxylic Acid-d5	25mg
	6	IS_MEIAA	d5-MEIAA	10mg
	7	IS_DZ	d3-DZ	1mg
	8	IS_cZ	15N4-cZ	1mg
	9	IS_iP9G	d6-iP9G	1mg
	10	IS_tZ	d5-tZ	1mg
	11	IS_BAP	d7-BAP	1mg
	12	IS_DHZOG	d7-DHZOG	2mg
	13	IS_IP	d6-IP	1mg
	14	IS_IPR	d6-IPR	1mg
	15	IS_GA4	d2-GA4	50ug
	16	IS_GA9	d2-GA9	50ug
	17	IS_GA53	d2-GA53	250ug
	18	IS_JA	d5-JA	10mg
	19	IS_MEJA	d5-MEJA	10mg
	20	IS_SA	d4-SA	0.1g
	21	IS_TRP	[2H5]-L-TRP	500mg
	22	IS_2MeStZR	[2H5]2-METHYLTHIO-trans-ZEATIN RIBOSIDE	1mg
	23	IS_tZOG	[2H5]trans-ZEATIN-O-GLUCOSIDE	1mg
	24	IS_DHZROG	[2H7]DIHYDROZEATIN-O-GLUCOSIDE RIBOSIDE	2mg
	25	IS_DHZR	[2H3]DIHYDROZEATIN RIBOSIDE	1mg
	26	IS_ICAld	Indole-3-carboxaldehyde-13C	5mg
	27	IS_IAA-Ala	[2H5]INDOLE-3-ACETYL-L-[15N] ALANINE	250ug
	28	IS_IAM	[2H5]-IAM	10mg
	29	IS_IAN	[2H4]INDOLE-3-ACETONITRILE	100ug
	30	IS_TRA	Tryptamine-α,α,β,β-d4 HCl	10mg
	31	IS_ILA	DL-Indole-3-lactic Acid-d5	1mg
	32	IS_IAA-Asp	[2H5]INDOLE-3-ACETYL-L-[15N] ASPARTIC ACID	250ug
	33	IS_GA19	[2H2]GIBBERELLIN A19	50ug
	34	IS_GA20	[2H2]GIBBERELLIN A20	250ug
